# Intercalated Language Degrees in Medicine

**DOI:** 10.15694/mep.2017.000014

**Published:** 2017-01-26

**Authors:** Fiona Field

**Affiliations:** 1 Imperial College London

**Keywords:** intercalated BSc, undergraduate education

## Abstract

This article was migrated. The article was marked as recommended.

## Letter

Intercalated Languages Degrees in Medicine

Language barriers in medicine can create many problems not limited to delayed diagnoses, obtainment of consent and how to relay information to back to the patient. As globalisation continues and hospitals begin to see more and more patients from abroad, I put forward the idea that medical schools should develop opportunities to study a language within their medical training.

At Imperial College London, students have the option to take evening classes in a language as part of the 'Horizons' course and similar courses exist elsewhere, however this is only sustainable in the younger years of medical school due to time constraints. If languages were offered as part of an intercalated degree, students would not be required to give up their free time to evening classes and hence there may be a larger uptake to the courses.

Many medical schools have already introduced the option of studying arts-based courses as an intercalated degree, such as Medical Humanities or Medical Journalism, however I think this could be developed further to include language degrees. This may require additional teaching staff and venues however many universities already offer undergraduate language degrees so this shouldn't be difficult to coordinate. Science and technology universities such as Imperial College may have more difficulties incorporating such a degree into the curriculum, however the recent introduction of Medical Humanities as an intercalated degree demonstrates that it is possible.

In summary, I believe opportunities to spend a year learning a language outside of general medical training may attract more students than current courses on offer and could significantly reduce the language barriers currently faced in medicine. 

## Notes On Contributors

Fiona Field

fiona.field12@imperial.ac.uk

Medical Student at Imperial College London

## Bibliography/References

## Declarations

The author has declared that there are no conflicts of interest.

