# Supplementary material for: Intercalated Language Degrees in Medicine
Source: MedEdPublish (2016). 2017 Jan 26;6:14. [Version 1] doi: 10.15694/mep.2017.000014 (PMC10885218; doi:10.15694/mep.2017.000014)
Supplement: Peer review history

# Reviewer report: Reviewer response for version 1

**DOI:** 10.21956/mep.19429.r28570

**Published:** 2017-05-20

**Authors:** Neel Sharma

**Affiliations:** 1 Albert Einstein College of Medicine, Montefiore Medical Center

This review has been migrated. The reviewer awarded 4 stars out of 5 As a nomad, I completely understand your POV. Medicine has become too streamlined so anything that doesn't fit within the box is not recognised or even understood. We are living in a connected world and patients as well as learners are pasture grazing. Hence an understanding of language and culture in a broader sense is now essential Keep fighting for this and you can make it happen

Reviewer Expertise:

NA

No decision status is available

# Reviewer report: Reviewer response for version 1

**DOI:** 10.21956/mep.19429.r28572

**Published:** 2017-01-30

**Authors:** Haipeng Xiao

**Affiliations:** 1 The First Affiliated Hospital, Sun Yat-sen University

This review has been migrated. The reviewer awarded 3 stars out of 5 This is an interesting letter promoting an opportunity of language learning during general medical training. The main focus is whether or not it should be compulsory and should it be even tackled during compulsory primary education prior to medical ones. Further relevant research will be worthwhile for the needs to be met based on current demographic characteristics in the country.

Reviewer Expertise:

NA

No decision status is available

# Reviewer report: Reviewer response for version 1

**DOI:** 10.21956/mep.19429.r28573

**Published:** 2017-01-27

**Authors:** Gert Van Zyl

**Affiliations:** 1 University of the Free State

This review has been migrated. The reviewer awarded 3 stars out of 5 This letter is a very interesting letter that brings back the issue of language as part of the formal curriculum. Living in a country with 11 official languages, the question of language being part of the formal curriculum, is a question that surface on a regular basis. We all know in health sciences the value of an excellent complete history in treating your patient. The secondary question when an institution decides to incorporate a language in the curriculum is if it must be based on a basic and medical terms basis only or a full version of the language. The dilemma of being fluent in the language means that one needs time and then there is the competition with the core curriculum. A letter that needs more thought and an excellent research question to look at curricula that have included language and those that have not included language and also to look at the spectrum of implementation of such language in curricula. Does the intervention (teaching a second language in medical context) have the impact on history taking, communication, patient care, patient satisfaction, rates of medico-legal issues and health outcomes, to mention a few. This letter needs to lead to research that is needed in this area of medical education.

Reviewer Expertise:

NA

No decision status is available

# Reviewer report: Reviewer response for version 1

**DOI:** 10.21956/mep.19429.r28574

**Published:** 2017-01-27

**Authors:** Thomas Puthiaparampil

**Affiliations:** 1 UNIVERSITI MALAYSIA SARAWAK

This review has been migrated. The reviewer awarded 2 stars out of 5 There is no dispute that knowledge of a patient's language is extremely important for efficient patient care. This proposal by Ms Fiona Field is worth considering. There is no issues if such courses are made optional and not compulsory.

Reviewer Expertise:

NA

No decision status is available

# Reviewer report: Reviewer response for version 1

**DOI:** 10.21956/mep.19429.r28571

**Published:** 2017-01-26

**Authors:** Trevor Gibbs

**Affiliations:** 1 AMEE

This review has been migrated. The reviewer awarded 3 stars out of 5 An interesting letter, which I think really touches on two aspects of the subject of intercalated degrees; their value and their content. Putting aside the question of their value and longevity in these days of financial restraint, their content is an interesting matter. To maintain any value there needs to be a wide picking list, chosen from a list of helpful and purposeful topics. If language is there, and I am sure there is a strong arguement that can be constructed around their positivity, then one wonders which language and whether it should be the medical language or the native tongue that is taught.I look forward to others comments on this letter

Reviewer Expertise:

NA

No decision status is available
